# Event and Comment

**Published:** 1920-06

**Authors:** 


### DENTAL REGISTER

THE MAGAZINE OF DENTISTRY

Vol. LXXIV | JUNE, 1920 | No. 6

### EVENT AND COMMENT

**The Sphere of the Dental Hygienist**

The profession of dentistry has discussed for several years, and with apparent lack of decision, the question of how the dental health problems can be managed to the best advantage. It is generally conceded, at this time that the professionally educated doctor of dental surgery is not being produced in sufficient numbers to supply the technical demands of the time, from the viewpoint of the profession. It has for some time been realized that we shall have to do something to increase the technical efficiency of our profession, or fail disastrously to meet the expectations of an enlightened public. The most hopeful suggestion at present is to train women to take on certain technical hygiene efforts which it is hoped will help to change the present tendency of bad teeth to a general betterment by prophylactic technique and better methods of instruction.

We have failed so far to agree unanimously that the dental hygienist is the most practicable functionary for our purpose, although a large number of the thoughtful members of the profession believe the efficient dental hygienist is at present the only way to meet the problem.

We have spent much valuable time discussing the feasibility of establishing such a functionary, and are in a fair way to begin the work. It must not be supposed that all the problems are cleared away and that all we have to do now is to begin the work, as we shall have to do some more legislating probably. We have enacted the necessary laws for less than a dozen states. Then we shall need to decide on a program of education for the hygienists, and this will be a large program and will need much machinery for putting it into effect. It is hardly possible it can be put into working condition at once, or easily, but we already have some of the fundamentals in the present dental college and state board organizations to serve as guides and precedents, and we have several institutions already that have made good progress in the development of instruction methods.

There are some problems for which we do not at present have recognized precedents, because their lines of work are not like those of the usual professional endeavors. For instance, the dental hygienist will not take on in a detailed way therapeutic functions to the extent pursued by the dentist. There will necessarily be some technical practice of a routine character; but there will also be much work of an instructive character that should be of immense value and very important from the viewpoint of dental health.

As this work is to be largely under the care of hygienists who are interested primarily in the health of children's teeth, it naturally follows that the work will deal specifically with those features of disease of children's teeth which are common and which offer means of treatment that will be most efficacious.

We are, as a matter of fact, entering upon a most important function, and it is only a large vision that will enable any one to sketch with a clear degree of adequacy the demands that will be made upon the dental hygienist when she has fully taken on her work.

The task is so big and so full of probabilities for great good that we have thought too lightly of it heretofore. We shall hope, however, to see it develop, slowly if need be, but rapidly if it can be, if only the great work becomes manifestly worth while. We are hoping a well-grounded basis of practice shall become established in due time and with the least amount of dissension.

